# Supplementary material for: Investigation of an Antioxidative System for Salinity Tolerance in Oenanthe javanica
Source: Antioxidants (Basel). 2020 Oct 1;9(10):940. doi: 10.3390/antiox9100940 (PMC7601823; doi:10.3390/antiox9100940)
Supplement: Supplementary file 1 [file antioxidants-09-00940-s001.zip › antioxidants-920004-Supplementary figures.pdf]

Supplementary figures

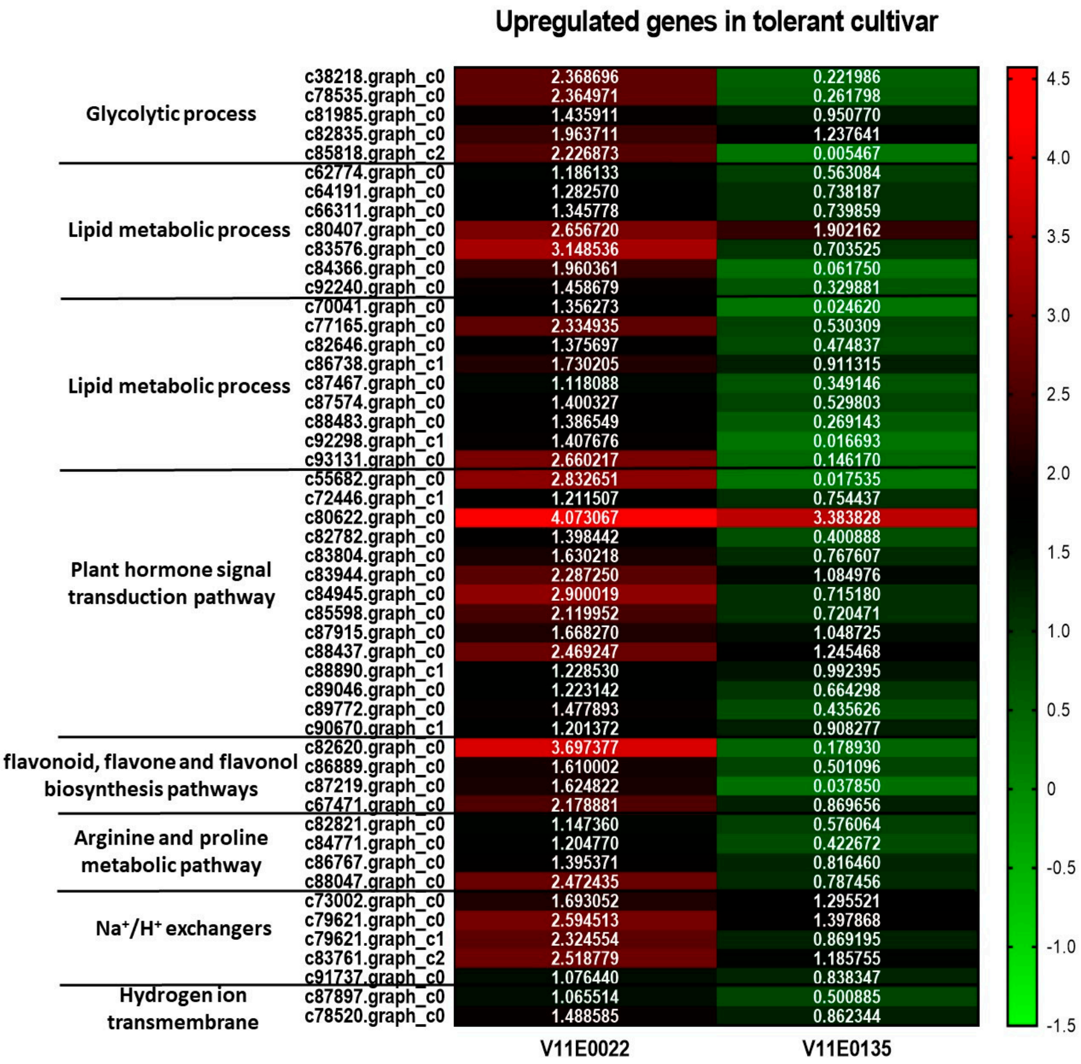

Supplementary figure 1. Heat maps of all upregulated DEGs in V11E0022 involved in different biological processes and pathways, and antiporters under salt stress conditions.

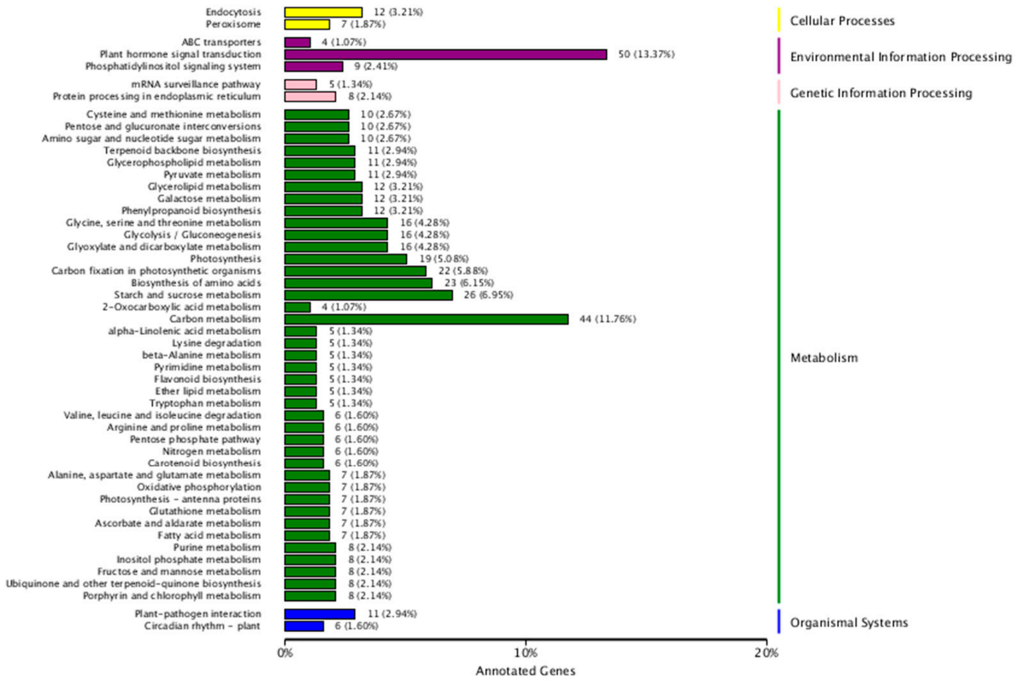

Supplementary figure 2. KEGG analysis of all DEGs in V11E0022 cultivar under salt stress treatment

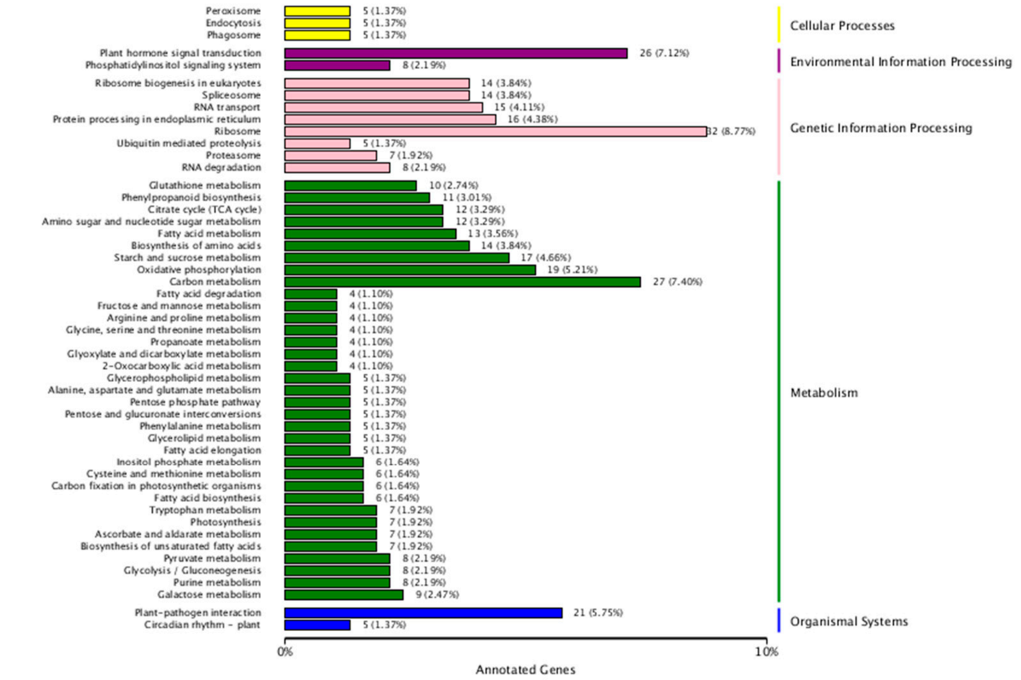

Supplementary figure 3. KEGG analysis of all DEGs in V11E0135 cultivar under salt stress treatment.

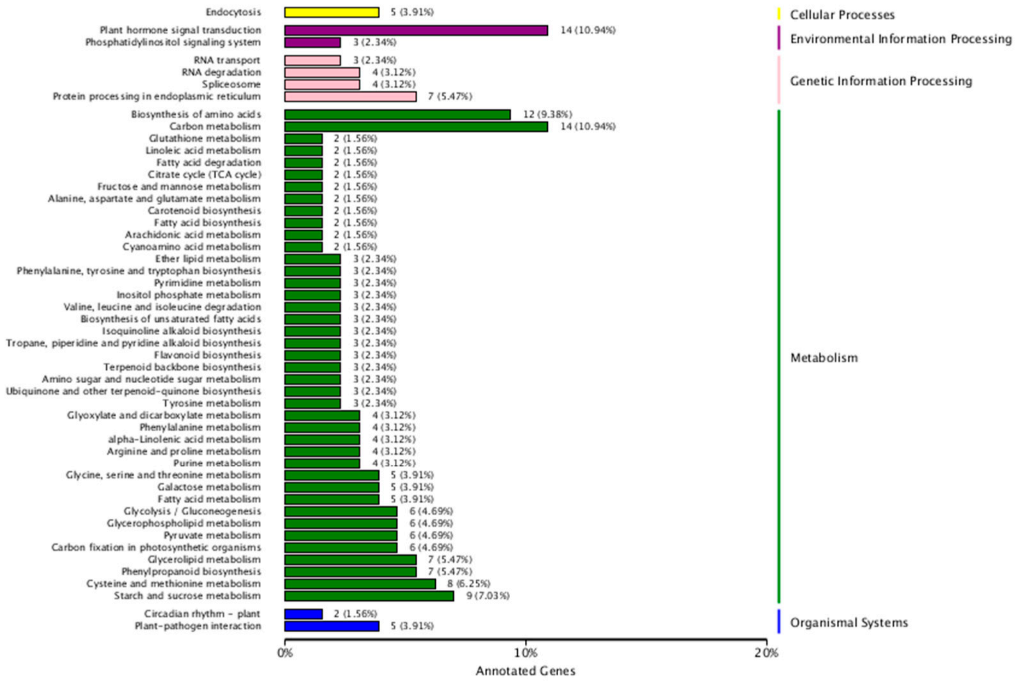

Supplementary figure 4. KEGG analysis of upregulated DEGs in V11E0022 cultivar under salt stress treatment.

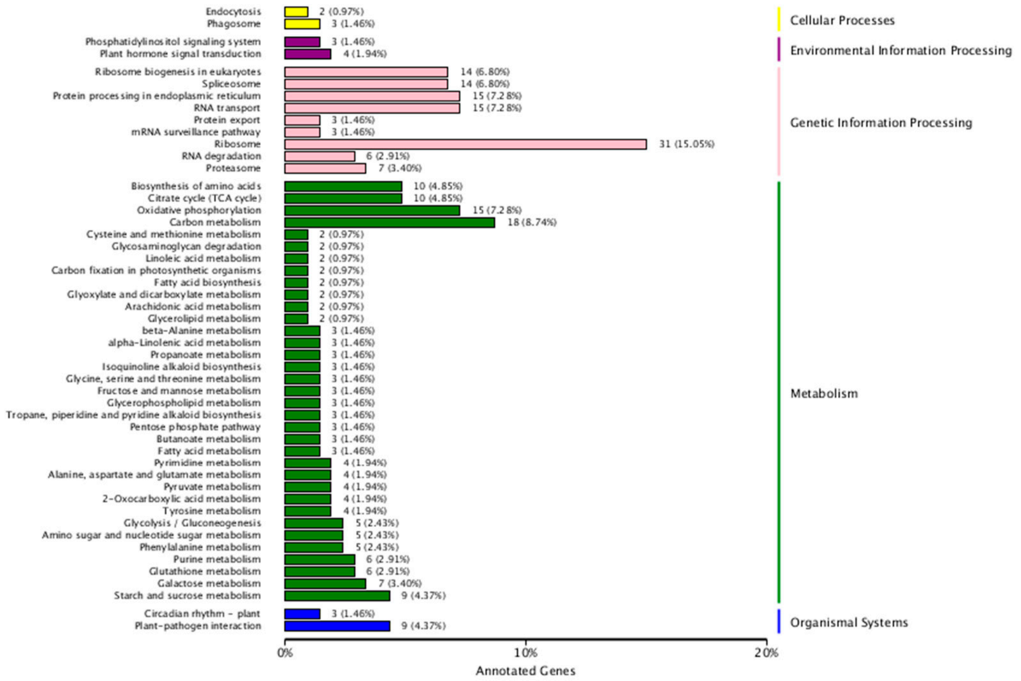

Supplementary figure 5. KEGG analysis of upregulated DEGs in V11E0135 cultivar under salt stress treatment.
